# Splenic Doppler Resistive Index Variation Mirrors Cardiac Responsiveness and Systemic Hemodynamics upon Fluid Challenge Resuscitation in Postoperative Mechanically Ventilated Patients

**DOI:** 10.1155/2018/1978968

**Published:** 2018-08-08

**Authors:** Claudia Brusasco, Guido Tavazzi, Chiara Robba, Gregorio Santori, Antonella Vezzani, Tullio Manca, Francesco Corradi

**Affiliations:** ^1^Anaesthesia and Intensive Care Unit, E.O. Ospedali Galliera, Genoa, Italy; ^2^University of Pavia, Department of Clinical, Surgical, Diagnostic and Pediatric Sciences. Intensive Care Unit, Fondazione Policlinico San Matteo IRCCS, Pavia, Italy; ^3^Anaesthesia and Intensive Care, San Martino Policlinico Hospital, IRCCS for Oncology, Genoa, Italy; ^4^Department of Surgical Sciences and Integrated Diagnostics, University of Genoa, Genoa, Italy; ^5^Cardiac Surgery, University of Parma, Parma, Italy

## Abstract

**Objective:**

To test if splenic Doppler resistive index (SDRI) allows noninvasive monitoring of changes in stroke volume and regional splanchnic perfusion in response to fluid challenge.* Design and Settin*g. Prospective observational study in cardiac intensive care unit.

**Patients:**

Fifty-three patients requiring mechanical ventilation and fluid challenge for hemodynamic optimization after cardiac surgery.

**Interventions:**

SDRI values were obtained before and after volume loading with 500 mL of normal saline over 20 min and compared with changes in systemic hemodynamics, determined invasively by pulmonary artery catheter, and arterial lactate concentration as expression of splanchnic perfusion. Changes in stroke volume >10% were considered representative of fluid responsiveness.

**Results:**

A <4% SDRI reduction excluded fluid responsiveness, with 100% sensitivity and 100% negative predictive value. A >9% SDRI reduction was a marker of fluid responsiveness with 100% specificity and 100% positive predictive value. A >4% SDRI reduction was always associated with an improvement of splanchnic perfusion mirrored by an increase in lactate clearance and a reduction in systemic vascular resistance, regardless of fluid responsiveness.

**Conclusions:**

This study shows that SDRI variations after fluid administration is an effective noninvasive tool to monitor systemic hemodynamics and splanchnic perfusion upon volume administration, irrespective of fluid responsiveness in mechanically ventilated patients after cardiac surgery.

## 1. Introduction

Fluid resuscitation is a cornerstone of perioperative management of patients undergoing cardiac surgery. The effects of extracorporeal circulation, together with the underlying cardiac disease, are often associated with substantial changes in intravascular volume and hemodynamic status, resulting from possible inflammation processes, hormonal influences, and pharmacological interactions. Hypovolemia and tissue hypoperfusion may occur after surgery and remain undetected, thus leading to postoperative complications, multiorgan dysfunction, prolonged hospital stay, and increased mortality [1–6]. On the other hand, there is evidence that excessive fluid administration may result in cardiac and systemic fluid overload, with negative impact on wound healing and intestinal peristalsis [7, 8]. Maintenance of adequate cardiac preload is considered as a primary target to optimize left ventricular (LV) performance and global oxygen delivery. Therefore, indexes reflecting cardiac preload and responsiveness to volume administration are of particular interest to the clinician. Animal studies [9, 10] suggest that the spleen may contribute to acute adjustments of cardiovascular function upon volume loading by several mechanisms, including blood reservoir function [9], fluid extravasation from splenic circulation into lymphatic reservoirs, and modulations of blood pressure by reflexes and neurohormonal mechanisms [10]. The role of the spleen in humans has not been yet extensively investigated. In clinical practice, the measurement of the spleen size is commonly used even if it represents only an indirect method to assess splenic circulation and its clinical application is limited by wide interindividual variability. Splenic Doppler resistive index (SDRI) can provide a more direct and less variable assessment of splenic circulation [11] without requiring estimates of Doppler angle or vessel cross-sectional area. Moreover, SDRI is independent of perfusion pressure and is increased by hypotension, hypovolemia, or anemia, and it has been shown to be a clinically useful noninvasive method for early detection of acute cardiovascular changes and persistent occult hypoperfusion in critically ill patients [12].

The aim of the present study was to explore the association between changes of splanchnic perfusion inferred by SDRI and changes of stroke volume in response to fluid challenge during mechanical ventilation following major cardiac surgery.

## 2. Materials and Methods

### 2.1. Patients

The study was approved by the Review Board of our University Hospital (Protocol no. 812/2014). Study protocol and aim were explained to patients before elective cardiac surgery and written informed consent was obtained. Fifty-three consecutive patients admitted to the intensive care unit after elective cardiac surgery were prospectively included (Table 1). No statistically significant differences between male and female in terms of age, simplified acute physiology score, and vasoinotropic score were present. The candidate patients were required to have a pulmonary artery catheter in place as per clinical indications, age >18 yr, absence of any condition known to modify SDRI (arrhythmia, aortic regurgitation or stenosis, intra-abdominal hypertension, or intra-aortic balloon pump), not to be under renal replacement therapies, or mechanical ventilation with positive end-expiratory pressure >5 cm H_2_O or fraction of inspired oxygen >50%.

### 2.2. Study Protocol

All patients were sedated with continuous infusion of propofol and mechanically ventilated with tidal volume of 6 mL/kg of predicted body weight. The eligible patients were studied when a fluid challenge was required to optimize hemodynamic status. SDRI measurements were obtained before fluid challenge (T0) and 5-10 min after 500 mL of normal saline had been centrally infused for 15–20 min by pressure bag (T1). Investigators who performed the SDRI measurements were not involved in patient care, and the physicians in charge of patient care were blinded to SDRI results. The following data were recorded before and after fluid challenge: stroke volume (SV), central venous pressure (CVP), mean arterial pressure (MAP), pulmonary artery occlusion pressure (PAOP), systemic vascular resistance index (SVRI), mixed venous oxygen saturation (SvO2), and arterial lactate level (cLac). Percentage changes between T0 and T1 were calculated. Patients were defined as fluid challenge responders when stroke volume at T1 was ≥10% than at T0.

### 2.3. Hemodynamic Monitoring

Patients were positioned 30° supine and all pressure transducers were referred to mid chest at the level of right atrium. All patients were monitored with invasive arterial blood pressure by radial (Arterial Leadercath 3F, Vygon, Ecouven, France) and pulmonary artery catheters (141HF7, Edwards Lifesciences, Unterschleißheim, Germany). Clinical data were collected from bedside monitors (Drager Infinity Delta XL, Drager Medical GmbH Lubeck, Germany). Cardiac output was measured by intermittent thermodilution with 10-mL normal saline injected into the superior vena cava via pulmonary artery catheter. Three consecutive injections were randomly performed during the respiratory cycle. When measurements differed by >10%, the cardiac output was further measured twice and the average value was calculated after exclusion of the highest and lowest values. In order to reduce interoperator variability, saline was always injected by the same physician. Thermodilution curves were retained if they showed stable baseline temperature, undisturbed rapid rise, and exponential decay without signs of early recirculation. Blood samples (2 mL each) were collected from the tip of pulmonary artery catheter to measure SvO_2_ (ABL800 FLEX, Radiometer Medical ApS, 2700 Brønshøj, Denmark). The correct positioning of the catheter was confirmed by the waveform of the pressure curve, catheter length, and a chest X-ray.

### 2.4. Splenic Doppler Resistive Index Measurements

Ultrasonographic examinations were obtained with a 2.5-MHz phase array transducer, provided with a color-pulsed wave Doppler device for studying of the spleen by last-generation US equipment (Philips CX50 system Philips Healthcare). Patients were placed in a supine position and, after a preliminary examination of abdominal cavity and organs, the complete spleen visualization was obtained in a coronal view including the hilum. Doppler US measurements were obtained by an anesthesiologist (F.C.) with 15 years of experience in Doppler ultrasound. The transducer was positioned on the left intercostal spaces. Color Doppler allowed identification of the main branches of the splenic artery, which were measured just past the hilum, at a distance of 1 cm from it, inside the spleen in a straight tract of the vessel, avoiding more peripheral vessels [13]. The sample gate was adjusted to the site of the vascular lumen. Gain and scale frequency were adjusted for optimum color signals. Waveforms were recorded, and SDRI was calculated as the ratio (S - D) / S, where S and D stand for peak systolic and end-diastolic velocities, respectively. To minimize sampling error, the pulsed wave Doppler spectrum was increased by using the lowest frequency shift range not causing aliasing, and the wall filter was set at a low frequency. Ultrasound examinations were considered technically adequate if they met the following criteria: (i) clear two-dimensional image with definition of spleen parenchyma; (ii) good color image with representation of the intrasplenic vascular blood flow; (iii) at least three Doppler time-velocity spectra representative of all components of arterial flow, from the early systolic to the end-diastolic Doppler shifts. Measurements were obtained over three splenic areas (upper, middle, and lower poles) and the average was retained as the SDRI for analysis.

### 2.5. Statistical Analysis

All results are expressed as means ± standard deviations, counts, percentages, odds ratio (OR), and 95% confidence interval (CI). The Shapiro-Wilk test was used to evaluate the normal distribution of continuous variables. Unpaired and paired Student's* t*-test were used for comparisons between and within groups, respectively. Fisher's exact test was used for comparisons of frequencies. A sample size of at least 51 patients was required for a paired* t*-test with power = 0.8, *α* = 0.05, effect size = 0.4, and a two-sided alternative hypothesis. Correlations were determined by Pearson's test. To assess the diagnostic accuracy of SDRI values in the prediction of an increase in SV of 10% or higher, sensitivity receiver operating characteristic (ROC) curves were constructed and the following parameters determined: sensitivity, specificity, positive predictive value (PPV), and negative predictive value (NPV).

The percentage changes between T0 and T1 of main hemodynamic and clinical variables were entered into univariate logistic regression models with responsiveness to fluid challenge as the dependent variable. The variables that reached statistical significance at univariate analysis were entered into a multivariate logistic regression model. The le Cessie-van Houwelingen-Copas-Hosmer test was used to assess the goodness-of-fit for each logistic regression model [14]. A two-sided* p* value <0.05 was assumed as statistically significant. Statistical analyses were performed by using SPSS software (version 20.0; SPSS Inc, Chicago, IL), GraphPad Prism 6.00 (GraphPad Software, San Diego, CA), and the R software/environment (version 3.4.2; R Foundation for Statistical Computing, Vienna, Austria).

## 3. Results

### 3.1. Baseline Conditions

Before fluid challenge, SDRI was not significantly different between responders and nonresponders (0.68 ± 0.09 and 0.64 ± 0.09; p = 0.106). The same was true for MAP (76 ± 10 versus 73 ± 12 mmHg; p = 0.371), PAOP (15 ± 6 versus 18 ± 6 mmHg; p = 0.155), SvO_2_ (59 ± 9 versus 63 ± 7 %; p = 0.054), and cLac (1.6 ± 0.9 versus 1.3 ± 0.6 mmol/L; p = 0.141). However, stroke volume (45 ± 14 versus 59 ± 20 mL; p = 0.007) and CVP (12 ± 4 versus 14 ± 4 mmHg; p = 0.041) were lower in responders than nonresponders, whereas SVRI was higher (2490 ± 821 versus 1928 ± 617 dyne-sec/cm^−5^/m^2^; p = 0.010).

### 3.2. Changes after Fluid Challenge

#### 3.2.1. Hemodynamics

After fluid challenge, 30 of the 53 patients included (57%) were fluid challenge responders. Stroke volume change after fluid challenge was 23±13% in responders and 5±3% in nonresponders (p<0.001). In responders, stroke volume (p<0.001) and SVO_2_ (p = 0.012) were significantly increased and SDRI (p<0.001) and SVRI (p<0.001) significantly decreased, while CVP, MAP, PAOP, and cLac did not change significantly. In nonresponders, stroke volume was also increased (p<0.001) while SDRI (p = 0.006) and cLac (p = 0.017) decreased significantly. No significant changes were observed for CVP, MAP, PAOP, SVRI, and SVO_2_ (Table 2).

#### 3.2.2. Splenic Doppler Resistive Index

**Figure 1:**
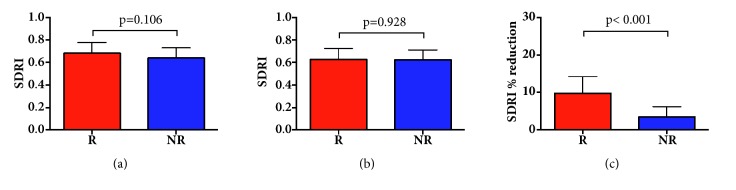
(a) Before fluid challenge, SDRI was not significantly different between responders (R) and nonresponders (NR). (b) After fluid challenge, SDRI was not significantly different between R and NR. (c) SDRI percent reduction was statistically different between responders (R) and nonresponders (NR).

**Figure 2:**
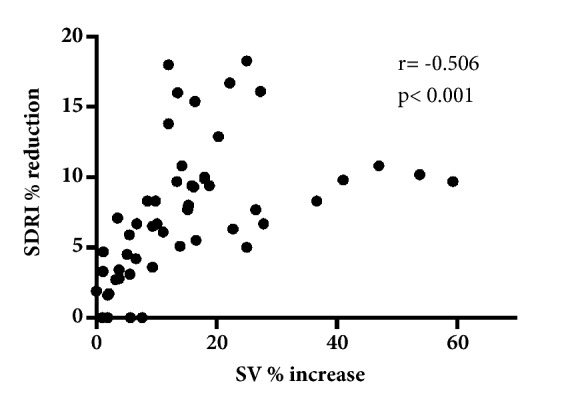
Stroke volume percent increase was significantly correlated with SDRI percentage decrease after fluid challenge.

**Figure 3:**
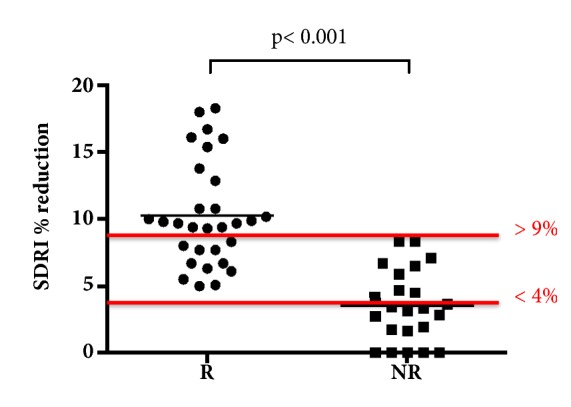
A SDRI percent change >9% was representative of fluid responsiveness, with 100% specificity, 100% positive predictive value, 63% sensitivity, and 68% negative predictive value. A SDRI percent change <4% excluded fluid responsiveness, with 100% sensitivity, 100% negative predictive value, 61% specificity, and 77% positive predictive value. None of the fluid challenge responders (R) had a SDRI percent change <4%, and conversely none of the fluid challenge nonresponders (NR) had a SDRI percent change >9%.

Absolute values of SDRI were significantly reduced both in responders (0.68 ± 0.09 before and 0.63 ± 0.09 after fluid challenge; p <0.001) and nonresponders (0.64 ± 0.09 before and 0.62 ± 0.09 after fluid challenge; p = 0.006; Table 2) without differences between groups. However, percentage reductions of SDRI were significantly greater (p <0.001) in responders (10 ± 4%) than nonresponders (3.5 ± 2.7%) (Figure 1). SDRI was nonsignificantly different in patients with and without vasopressors before (0.62 ± 0.09 versus 0.67 ± 0.09, respectively; p = 0.131) and after fluid challenge (0.58 ± 0.07 versus 0.63 ± 0.09, respectively; p = 0.097). In addition, no statistically significant difference was found between SDRI percent reduction and vasopressors (7.6% in patients with vasopressors versus 7.3% in patients without vasopressors; p=0.841). SDRI percent reduction was significantly correlated with stroke volume change after fluid challenge (Figure 2) in the whole population (*r *= –0.51; p<0.001). The area under the ROC curves (AUC) was significant for SDRI percent reduction (AUC: 0.88, 95% CI: 0.80-0.96; p<0.001). A SDRI reduction >9% was a marker of fluid responsiveness, with 100% specificity, 100% positive predictive value, 63% sensitivity, and 68% negative predictive value. A SDRI reduction <4% excluded fluid responsiveness, with 100% sensitivity, 100% negative predictive value, 61% specificity, and 77% positive predictive value. No responder had SDRI reductions < 4%, and conversely none of the nonresponders had SDRI reductions >9% (Figure 3).

Twenty patients had SDRI reduction between 4% and 9%. Eleven of them were responders and 9 nonresponders. In the former, cLac significantly decreased from 1.8 ± 1 mmol/L before fluid challenge to 1.3 ± 0.6 mmol/L after fluid challenge (p = 0.022) and SVRI from 2663 ± 1089 before fluid challenge to 2175 ± 780 dyne-sec/cm^−5^/m^2^ after fluid challenge (p = 0.027). By contrast, in nonresponders only cLac significantly decreased from 1.5 ± 0.6 mmol/L before fluid challenge to 1.2 ± 0.5 mmol/L after fluid challenge (p = 0.009), while SVRI was insignificantly changed from 1893 ± 628 before fluid challenge to 1739 ± 545 dyne-sec/cm^−5^/m^2^ after fluid challenge (p = 0.184).

Patients with SDRI reduction <4% showed no further reduction in cLac levels (from 1.2 ± 0.48 mmol/L before fluid challenge to 1.2 ± 0.47 mmol/L after fluid challenge; p = 0.161) or SVRI (from 1953 ± 633 before fluid challenge to 1885 ± 547 dyne-sec/cm^−5^/m^2^ after fluid challenge; p = 0.551)

#### 3.2.3. Perfusion

SDRI measurements were statistically correlated with stroke volume (*r* = –0.37; p = 0.007), cLac (*r* = –0.45; p = 0.001) and SVO_2_ (*r* = –0.52; p <0.001) before and after fluid challenges.

Fluid challenge caused an increase of SDRI ≥4% in 39 patients but a stroke volume increase ≥10% only in 30 of them (p < 0.001). The occurrence of SDRI percent reduction >4% was 100% in responders and 39% in nonresponders. The percent increase of stroke volume was significantly correlated with the percent decrease of SDRI in the whole population (*r* = –0.51; p <0.001). However, this correlation was significant in nonresponder (*r* = –0.55; p <0.007) but not in responder (*r* = -0.23; p <0.905) group. In 9 out of 23 patients with a stroke volume increase <10% after fluid challenge there was a decrease of cLac from 1.4 ± 0.8 to 1.2 ± 0.6 mmol/L (p = 0.017), and SDRI from 0.67 ± 0.12 to 0.63 ± 0.11 (p <0.001), suggesting an improvement in splanchnic perfusion.

### 3.3. Logistic Regression Model

**Figure 4:**
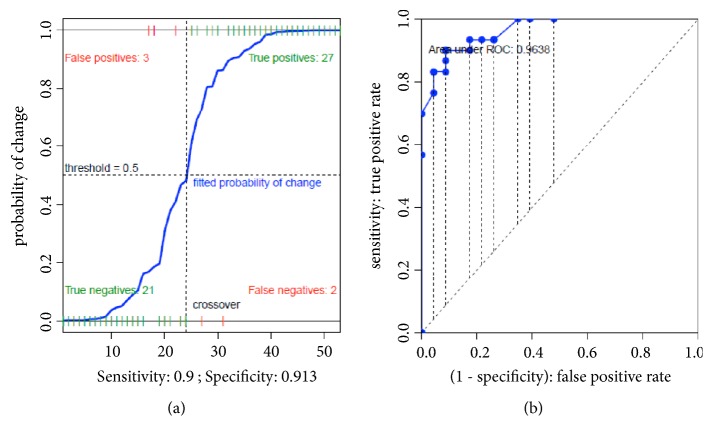
(a) Sensibility and specificity of the multivariate logistic regression model for fluid challenge responsiveness (SDRI, p=0.001; SVRI, p=0.163; SVO_2_, p=0.159). (b) ROC curve with the area under curve (AUC) of the same multivariate logistic regression model.

The main hemodynamic and clinical parameters calculated as percentage change between T0 and T1 were entered into univariate logistic regression models, assuming responsiveness to fluid challenge as dependent variable (Table 3). The variables that reached statistical significance at univariate analysis were then entered into a multivariate logistic regression model. Of these latter, only SDRI percent reduction was statistically significant (p = 0.001; Table 3). The multivariate model showed an excellent performance for both sensitivity and specificity (Figure 4(a)), with an AUC = 0.963 (Figure 4(b)).

## 4. Discussion

The main findings of our study were that (1) an SDRI reduction >9% was a marker of fluid responsiveness, with 100% specificity and 100% positive predictive value; (2) an SDRI reduction >4% was always associated with an improvement of splanchnic hypoperfusion mirrored by an increase in lactate clearance and reduction in systemic vascular resistance, regardless of fluid responsiveness; and (3) a SDRI reduction <4% excluded fluid responsiveness, with 100% sensitivity and 100% negative predictive value, without improvement in splanchnic perfusion.

Stukely firstly suggested in 1722 that the spleen was a “diverticulum of the systemic circulation, filling and empting with blood, thus acting as a controller of blood volume” [15]. In animals, the spleen constitutes an integral part of the splanchnic vasculature and receives about 10% of cardiac output; this peculiar anatomical and physiological organization optimally position it as a mirror of intravascular volume. Available evidence suggests that mobilization of blood from the splanchnic region during hypovolemia and pooling of infused fluid occur via a reflex regulation involving atrial receptors and sympathetic innervation of splanchnic capacitance vessels [16]. Moreover, the spleen is prone to venous congestion, thus regulating venous return. These observations suggest a role for the spleen as a window to the splanchnic circulation. The present human study confirms the spleen as a precisely regulated reservoir responding to hemodynamic variations induced by fluid challenge. The large variability of spleen volume in adult humans makes the simple evaluation of spleen size unreliable for hemodynamic assessment. The only parameter validated and described in various contexts for this purpose is the SDRI because it is easier to measure and shows a better defined diastolic phase if compared with other splanchnic arteries (i.e., superior mesenteric artery) [11, 17, 18]. In addition, SDRI provides information on downstream arterial vascular resistance, with the consequent possibility of detecting hemodynamic abnormality related to organ dysfunction before biochemical and macrohemodynamic changes. The upper limit of normality of SDRI has been established to be <0.6 [13]. In critically ill patients SDRI may variably increase, depending on clinical condition and multiple factors (amines, transfusions, respiratory exchanges, mechanical ventilation, etc.). Therefore, percent changes in SDRI can be more clinically informative than its absolute values.

The reduction of arterial lactate concentration observed in nonresponder patients to fluid challenge confirms previous experimental data, suggesting [19] that splanchnic perfusion may improve in response to administered fluid load even when stroke volume changes are considered insignificant. Moreover, our results indicate that direct assessment of splanchnic perfusion with SDRI may be advantageous for monitoring systemic effects of fluid resuscitation, because patients not responding to fluid challenge reduce arterial lactate concentration when SDRI is reduced by >4%. Yet, because there was no difference in lactate clearance across groups, it is possible that the reduction in SDRI in responders was mainly due to changes in venous compliance rather than improved splanchnic arterial perfusion. Indeed, venous return, which is one of the most important determinants of stroke volume, can be modified by changes of either intravascular volume or vascular compliance.

A number of indexes that allow predicting fluid responsiveness have been already proposed [20], but their use is limited by the need of deep sedation and lack of validation during protective mechanical ventilation [21, 22]. On the contrary, SDRI measurements do not require sedation and are feasible also in spontaneously breathing patients. Moreover, SDRI allows estimating splanchnic perfusion even when, as in patients with cardiac surgery, the Frank-Starling mechanism may not be applied due to myocardial dysfunction.

### 4.1. Incremental Value of SDRI

Our study has a number of strengths. For the first time, it describes the relationship between invasive stroke volume and noninvasive SDRI after fluid administration in the postoperative period. Furthermore, our results provide evidence that improvement in splanchnic circulation occurs in either the presence or absence of stroke volume responsiveness, and SDRI percent reduction is representative of active vasodilation in the splanchnic circulation, as confirmed by its correlation with cLac and SVO_2_. Therefore, if fluid administration is exclusively based on changes in stroke volume, the opportunity to revert ongoing vasoconstriction and improving splanchnic perfusion might be missed.

### 4.2. Limitations

Our study has several limitations. First, it was not designed to evaluate the influence of confounding factors, such as age and abnormal arterial stiffness, which may have affected SDRI. Second, in this study only normal saline was used for fluid challenge, which makes the results not directly applicable to conditions where different fluids are infused or in patients receiving nutritional supplementation. Third, our recording period was relatively short because the aim was to study acute physiological responses to fluid challenge, thus possibly missing late physiological responses due to fluid balance restoration or fluid redistribution. Fourth, the study was not powered to assess differences in clinical outcomes. Fifth, inflammation and ensuing increases in splenic blood flow could possibly be a potential confounding factor, especially in cardiac surgery patients due to CEC procedure.

## 5. Conclusion

Significant changes in splenic Doppler resistive index assessed with Doppler ultrasonography mirror significant hemodynamic variations induced by fluid challenge and allow a valuable and repeatable bedside method to assess splanchnic perfusion regardless of fluid responsiveness.

## Figures and Tables

**Table 1:** Baseline characteristics at inclusion in 53 patients and type of surgery.

Age (years)	72±8
Sex, m/f	41/12
Simplified Acute Physiology Score II	29±11
Euroscore	7±3
Hypertension	50
Diabetes	13
Dyslipidemia	22
Extracorporeal circulation duration (min)	32±1
Vaso-Inotropic score	22±14
Coronary artery by-pass grafting (n)	24
Valve Surgery (n)	11
Mixed (n)	11
Bentall procedure (n)	7

**Table 2:** Hemodynamic findings before and after fluid challenge.

**Parameters**	**FC Responders (*n=30*)**			**FC Non-responders (*n=23*)**		
	**Before FC**	**After FC**	***P value***	**Before FC**	**After FC**	***P value***
SV (mL)	45±14	59±18	<0.001	59±20	61±20	<0.001
SDRI	0.68±0.09	0.63±0.10	<0.001	0.64±0.09	0.62±0.09	0.006
CVP (mmHg)	12±4	12±4	0.554	14±4	13±4	0.200
MAP (mmHg)	76±10	75±8	0.609	73±12	72±8	0.933
PAOP (mmHg)	15±6	15±7	0.998	18±6	16±4	0.118
SVRI (dyne-sec/cm^−5^/m^2^)	2490±821	1935±575	<0.001	1928±617	1825±538	0.195
SVO_2_ (%)	58±9	63±7	0.012	63±7	63±6	0.686
cLac (mmol/L)	1.6±0.9	1.4±0.7	0.108	1.4±0.8	1.2±0.6	0.017

FC: Fluid challenge; SV: Stroke Volume; SDRI: Splenic Doppler resistive index; CVP: Central Venous Pressure; MAP: Mean artery pressure; PAOP: pulmonary artery occlusion pressure; SVRI: systemic vascular resistive index; SVO_2_: mixed venous oxygen saturation; cLac: arterial lactate.

**Table 3:** Univariate and multivariate logistic regression analysis for fluid challenge responsiveness.

**Univariate Logistic Regression**			
**Variable**	**RC**	**OR (95**%** CI)**	***P value***
SDRI, %change	0.818	2.267 (1.569-3.989)	<0.001
CVP, %change	0.014	1.014 (0.980-1.054)	0.434
MAP, %change	-0.018	0.981 (0.907-1.061)	0.641
PAOP, %change	0.009	1.009 (0.973-1.051)	0.631
SVRI, %change	-0.035	0.964 (0.936-0.988)	0.009
SVO_2_, %change	0.129	1.138 (1.034-1.310)	0.029
cLac, %change	- 0.002	0.997 (0.982-1.011)	0.741

**Multivariate Logistic Regression**			
**Variable**	**RC**	**OR (95**%** CI)**	***P value***

SDRI %change	0.890	2.436 (1.577-4.845)	0.001
SVRI %change	-0.035	0.965 (0.912-1.009)	0.163
SVO_2_ %change	0.134	1.144 (0.996-1.467)	0.159

RC: regression coefficient; OR: odds ratio; CI: confidence interval; SDRI: splenic Doppler resistive index; CVP: central venous pressure; MAP: mean artery pressure; PAOP: pulmonary artery occlusion pressure; SVRI: systemic vascular resistive index; SVO_2_: mixed venous oxygen saturation; cLac: arterial lactate.

## Data Availability

Data supporting the present manuscript results are stored in a database and will be available if requested.
